# Identification of the Active Principle Conferring Anti-Inflammatory and Antinociceptive Properties in Bamboo Plant

**DOI:** 10.3390/molecules26103054

**Published:** 2021-05-20

**Authors:** Bruna Araujo Sousa, Osmar Nascimento Silva, William Farias Porto, Thales Lima Rocha, Luciano Paulino Silva, Ana Paula Ferreira Leal, Danieli Fernanda Buccini, James Oluwagbamigbe Fajemiroye, Ruy de Araujo Caldas, Octávio Luiz Franco, Maria Fátima Grossi-de-Sá, Cesar de la Fuente Nunez, Susana Elisa Moreno

**Affiliations:** 1 Programa de Pós-Graduação em Ciências Genômicas e Biotecnologia, Universidade Católica de Brasília, Brasília CEP 70790-160, DF, Brazil; soubrunabio@gmail.com (B.A.S.); williamfp7@yahoo.com.br (W.F.P.); ocfranco@gmail.com (O.L.F.); fatimasa@cenargen.embrapa.br (M.F.G.-d.-S.); 2 S-Inova Biotech, Programa de Pós-Graduação em Biotecnologia, Universidade Católica Dom Bosco, Campo Grande CEP 79117-900, MS, Brazil; osmar.silva@catolica.edu.br (O.N.S.); analeal.biotec@gmail.com (A.P.F.L.); dfbuccini@gmail.com (D.F.B.); caldasruy@gmail.com (R.d.A.C.); 3 Centro Universitário de Anápolis, Unievangélica, Anápolis CEP 75083-515, GO, Brazil; jfajemiroye@gmail.com; 4 Porto Reports, Brasília CEP 72236-011, DF, Brazil; 5 Embrapa Recursos Genéticos e Biotecnologia (Cenargen), Brasília CEP 70770-917, DF, Brazil; thales.rocha@embrapa.br (T.L.R.); lucianopaulinosilva@gmail.com (L.P.S.); 6 Núcleo de Estudos e Pesquisas Tóxico-Farmacológicas, Universidade Federal de Goiás, Goiânia 74605-220, GO, Brazil; 7 Departamento de Patologia Molecular, Faculdade de Medicina, Universidade de Brasília, Brasília CEP 70910-900, DF, Brazil; 8 Research Laboratory of Electronics, Massachusetts Institute of Technology, Cambridge, MA 02139, USA; cfuente@mit.edu; 9 Department of Biological Engineering, The Center for Microbiome Informatics and Therapeutics, Massachusetts Institute of Technology, Cambridge, MA 02139, USA; 10 Department of Electrical Engineering and Computer Science, The Center for Microbiome Informatics and Therapeutics, Massachusetts Institute of Technology, Cambridge, MA 02139, USA

**Keywords:** medicinal plants, natural products, folk medicine, neutrophil migration, pain and thaumatin

## Abstract

Early plants began colonizing earth about 450 million years ago. During the process of coevolution, their metabolic cellular pathways produced a myriad of natural chemicals, many of which remain uncharacterized biologically. Popular preparations containing some of these molecules have been used medicinally for thousands of years. In Brazilian folk medicine, plant extracts from the bamboo plant *Guadua paniculata* Munro have been used for the treatment of infections and pain. However, the chemical basis of these therapeutic effects has not yet been identified. Here, we performed protein biochemistry and downstream pharmacological assays to determine the mechanisms underlying the anti-inflammatory and antinociceptive effects of an aqueous extract of the *G. paniculata* rhizome, which we termed *AqGP*. The anti-inflammatory and antinociceptive effects of *AqGP* were assessed in mice. We identified and purified a protein (AgGP), with an amino acid sequence similar to that of thaumatins (~20 kDa), capable of repressing inflammation through downregulation of neutrophil recruitment and of decreasing hyperalgesia in mice. In conclusion, we have identified the molecule and the molecular mechanism responsible for the anti-inflammatory and antinociceptive properties of a plant commonly used in Brazilian folk medicine.

## 1. Introduction

Inflammation is a mechanism of host-defense that mounts local or generalized responses to confer protection against harmful stimuli such as injury or invasion by microbial pathogens. One such response is pain, which alerts the body that an injury has occurred [1]. However, this defense mechanism can itself become harmful to the host, causing severe tissue damage and potentially pain, resulting from chronic inflammation [2]. In addition to the host-protective role of neutrophils through phagocytosis and antimicrobial activity, these cells may exhibit deleterious functions [3,4]. Neutrophils can migrate to tissues and release lytic factors, reactive oxygen species, and other pro-inflammatory mediators, contributing to organ damage and chronic inflammation [5]. Chronic inflammation is a consequence of an exacerbated inflammatory response and is typically treated with anti-inflammatory drugs [6]. The most widely prescribed anti-inflammatory drugs are the nonsteroidal anti-inflammatory drugs (NSAIDs), of which non-selective NSAIDs are the oldest and most frequently prescribed worldwide [7]. The most common side effects of these drugs include gastrointestinal erosions and renal and hepatic failure [7,8]. Therefore, it is necessary to discover novel drugs against chronic inflammation that have fewer adverse effects [9].

Ethnopharmacological reports have demonstrated the use of plant extracts for the treatment of inflammatory disease and pain [10,11]. Indeed, many of these compounds produced by plants have been used in folk medicine for thousands of years. Early plants began colonizing earth about 450 million years ago and have exploited their versatile metabolism to produce numerous natural products that may serve as medicines [12]. One commonly used strategy to identify new drugs involves exploring the chemistry of folk medicinal plant extracts [13]. Among these natural products, peptides and proteins have been described that interact with animal systems, including the uterotonic cyclotides [14], immunomodulatory peptides [15,16,17,18,19], and anti-inflammatory thaumatin proteins [20,21].

Medicinal chemistry campaigns performed on plant extracts enabled the discovery of new anti-inflammatory compounds that are currently used in medicine [22,23,24]. In natural product discovery, medicinal chemistry is also key to ensure that any novel agent derived from extracts displays the appropriate safety and efficacy profiles [25,26]. Safety validation programs also stimulate the utilization of these plant extracts in public health assistance programs. In Brazil, the “*Living Pharmacies Project*” [27] has carried out ethnobotanical studies of more than 500 species, based on both folk knowledge and bibliographical research. These plants were cultivated in small units, or “*Living Pharmacies*”, each consisting of a medicinal vegetable garden and a workshop with the capacity to serve, free of charge, a community of 2000 to 3000 families [27]. Among the plants used in the project, ten species were found to provide therapeutic effects for 80% of the most common diseases in the area, including cutaneous and mucosal infections, digestive and respiratory diseases, rheumatic pain, and intestinal parasitic infections [27].

Here, we investigated the plant species *Guadua paniculata* Munro *paniculata* (Poaceae, subfamily Bambusoideae) as a potential source of novel anti-inflammatory agents. Isolated from the Brazilian Cerrado and used as part of the *Living Pharmacies Project*, this species has long been used in folk Brazilian medicine for the treatment of pain, thus pointing towards a potential anti-inflammatory effect. Several species from the Bambusoideae subfamily have been reported to have therapeutic properties for the treatment of hypertension, obesity, paralysis, sweating, and inflammation [28,29,30]. The present study evaluated aqueous extracts from the *G. paniculata* rhizome (*AqGP*) for anti-inflammatory activity and toxicity towards human cells and identified the compound responsible for its pharmacological action.

## 2. Results and Discussion

**Figure 1:**
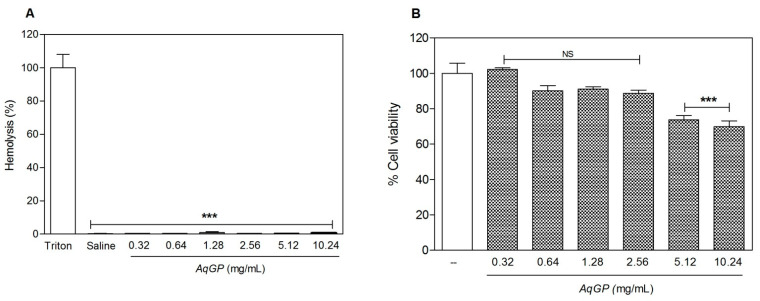
Effects of *AqGP* in vitro against goat erythrocytes and murine cell line NIH/3T3. (**A**) The hemolytic activity of *AqGP* on goat erythrocytes was evaluated. (**B**) The effect of *AqGP* on cell viability of the cell line NIH/3T3 was evaluated by MTT assay. *** *p* < 0.05 difference as compared to the untreated group, which received only water (ANOVA followed by Bonferroni’s test); NS: not significant.

Validation of the safety of plant extracts is an essential part of evaluating the effectiveness of their pharmacological properties. To evaluate *AqGP* toxicity, two in vitro methodologies were used: hemolytic activity towards caprine cells and effects on cell viability using murine and human cells. Cell viability was evaluated at six concentrations of *AqGP* (0.32, 0.64, 1.28, 2.56, 5.12, and 10.24 mg/µL). The results showed a very low percentage of hemolysis (0.66%) at all the tested concentrations (Figure 1A). In MTT assays, when murine fibroblasts (NIH/3T3 cell lines) were exposed to these concentrations of *AqGP* (0.32, 0.64, 1.28, 2.56, 5.12, and 10.24 mg/µL), the viability of the cell line NIH/3T3 was reduced by 26% and 30%, when cells were exposed to the higher doses tested (5.12 and 10.24 mg/mL, respectively) (Figure 1B). The same procedure was performed using human breast cancer MCF-7 cells, for which no reduction in viability was observed even at the highest *AqGP* concentrations tested (10.24 mg/mL) (data not shown). Given the absence of cytotoxic effects towards mammalian cells, extensive in vivo experiments were performed. The harmful concentration of *AqGP* observed was approximately four times higher than the active one, demonstrating a comfortable safety margin between the active and harmful doses of the molecules. Despite the possible allergenic epitope in thaumatin, these results indicate that using the extract in question is safe in practice [31,32].

**Figure 2:**
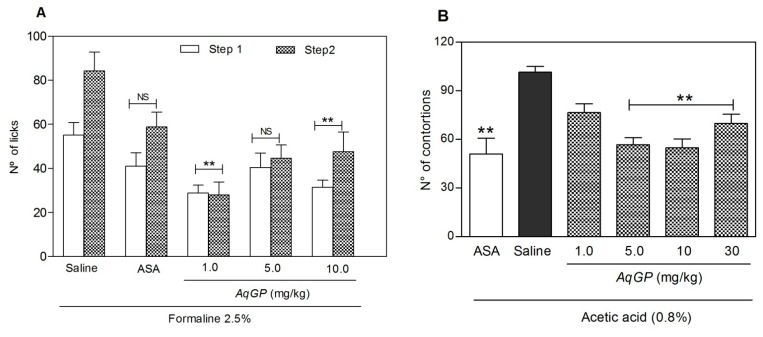
Effect of *AqGP* pre-treatment on hyperalgesia in mice. All animals were pre-treated with the *AqGP* extract at concentrations of 1, 5, or 10 mg/kg or acetylsalicylic acid (100 mg/kg) 15 min after the injection of nociceptive stimulant. (**A**) Hyperalgesia was induced by formalin (20 µL, 2.5%) in the sub-plantar region of the right paw. The number of licks was counted in two phases, phase 1 from 0 to 5 min, and phase 2 from 15 to 30 min. (**B**) Hyperalgesia induced by acetic acid (0.8%, 100 μL, i.p). The frequency of abdominal contortions was counted for 30 min. The results are expressed as the mean ± SEM of 5 animals. ** *p* < 0.05 when compared to the untreated control group (ANOVA followed by Bonferroni test). NS: not significant.

Evaluation of the anti-nociceptive response was performed using formalin and contortion tests [33]. Compounds that act on the first phase (phase 1) are characteristic of drugs with central analgesic activity. On the other hand, compounds that act on the later phase (phase 2) are characterized by the development of a local inflammatory process, indicating a peripheral analgesic activity generated by mediators released during inflammation that sensitize nociceptors [34]. In phase 1, the subcutaneous administration of *AqGP* at a concentration of 1.0 mg/kg of the extract showed significant inhibition (48%) of nociception, when compared with the treatment with formalin in phase 1 (after 0 to 5 min of the nociceptive stimulation; Figure 2A). In phase 2 (after 15 and 30 min of the nociceptive stimulation), at doses of 1.0, 5.0, and 10 mg/kg, mice exhibited a significant reduction (2.5%) in the frequency of paw-licking after injection of 20 µL of formalin (Figure 2A). Inhibition of nociception ranged from 43 to 67% for treated animals compared with animals that received only the noxious stimulus. By comparison, treatment with acetylsalicylic acid (ASA) (i.e., a drug with proved analgesic activity) at a dose of 100 mg/kg inhibited nociception by only 30% at the same stage. In order to confirm the peripherical analgesic effect of *AqGP*, the mouse writhing test was performed (Figure 2B). Similar to results observed in the formalin test, subcutaneous administration of *AqGP* at a concentration of 1.0 mg/kg of the extract showed a reduction (25%) of nociception when compared with the control treatment with acetic acid. At the concentrations of 5.0 and 10 mg/kg, *AqGP* as was able to inhibit the frequency of contortions (50%) for treated animals compared with mice that received only the noxious stimulus (Figure 2B). This result was similar to that observed in mice treated with acetylsalicylic acid.

**Figure 3:**
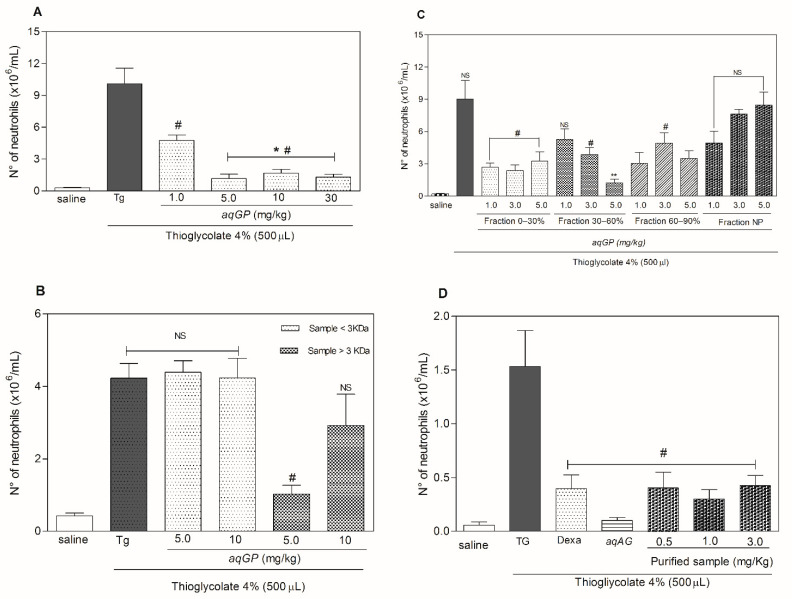
Anti-inflammatory bioassay-guided fractionation of *AqGP*. (**A**) Neutrophil migration to the peritoneal cavity in mice pre-treated with *AqGP*. (**B**) The migration of neutrophils to the peritoneal cavity was evaluated for mice pre-treated with fractions of *AqGP* collected from the ammonium sulfate precipitation process. (**C**) The migration of neutrophils to the peritoneal cavity in mice pre-treated with the peak three fraction (retention 29.9 min) obtained from fractionation by reverse phase chromatography of the 0–30% saturation fraction by ammonium sulfate of *AqGP*. (**D**) Anti-inflammatory activity after rechromatography in a hydrophobic affinity column of C4. Each experimental group had *n* = 5. Results are expressed as mean ± SEM of the number of neutrophils per mL. # *p* < 0.05 difference as compared to the untreated group injected with thioglycolate; * *p* < 0.05 difference as compared to the group treated with *aqGP* 1.0 mg/kg; ** *p* < 0.05 difference as compared to the group treated with *aqGP* fraction 0–30% (ANOVA followed by Bonferroni’s test); NS: not significant.

The anti-inflammatory properties of *AqGP* were analyzed by testing neutrophil migration to the peritoneal cavity of mice with peritonitis induced by injection of 4% thioglycolate. The use of irritants such as thioglycolate characteristically leads to the influx of neutrophils to the injured tissue, one of the key features of the inflammatory process [35]. Thus, thioglycolate has been extensively used as an efficient method to induce inflammation [36]. As observed in Figure 3A, the subcutaneous administration of *AqGP* at doses of 1.0, 5.0, 10, and 30 mg/kg administered subcutaneously in mice significantly reduced neutrophil migration to the intraperitoneal cavity (where inflammation had been induced by 4% thioglycolate, see methods). When treatment using thioglycolate (positive control) was compared to the negative control, the inhibitory activity of *AqGP* ranged from 53 to 88%. The extract modestly decreased neutrophil migration at 1.0 mg/kg^−1^ and exhibited its highest inhibitory potential at 5.0 mg/kg. The concentrations used in this assay showed no significant differences between their inhibition levels, indicating that increasing the concentration of *AqGP* does not enhance its inhibition potential. Although the tests performed in this study did not clearly delineate the mechanism of action of *AqGP*, it is plausible that the concentrations used lie within a range of saturation in which the active compound concentration does not lead to a progressive increase in activity (Figure 3).

The *AqGP* in the second phase demonstrated a peripheral analgesic effect, by which it would act on the inflammatory mediators that lead to pain. Consistent with these data, the acetic acid-induced abdominal contortions assay, a model of visceral pain [37], showed that all doses of *AqGP* were able to inhibit the nociceptive response in mice. Those data corroborate the anti-inflammatory activity demonstrated by the neutrophil migration test and are in agreement with the hypothesis that the use of *AqGP* in folk medicine is due to its activity to reduce inflammatory pain. In fact, separately analyzing the action of *AqGP* at a concentration of 1 mg/kg in both phases of the test, we observed a different behavior from other concentrations. The concentration of 1 mg/kg showed similar compounds that act on the central nervous system and are active in both phases. However, other tests that allow the evaluation of compounds on the central nervous system, such as the hot-plate test, should be conducted to further clarify this activity. The drug ASA used as a reference in the assay behaved as expected in this study, only acting on the second stage as it exerted a peripheral analgesic action [38].

Therefore, in order to evaluate and identify the main components responsible for the pharmacological activity of *AqGP*, the extract was purified and at each purification step, biological assays were performed. For each fractionation step, the most active fractions were selected for further purification steps (Figure 3). After dialysis, the internal dialysate containing molecules larger than 3.5 kDa exhibited an anti-inflammatory activity, inhibiting neutrophil migration by 75% when compared to the negative control, at a concentration of 5 mg/kg (Figure 3C). The external dialysate, however, was not active. The active internal dialysate sample was then precipitated by saturation at 0–30%, 30–60%, and 60–90% with ammonium sulfate. At all tested doses (1, 3, and 5 mg/kg), the fraction 0–30% presented activity and the percentage of inhibition varied between 60 and 71% (Figure 3B). The fraction with a saturation of 30–60% demonstrated activity only at concentrations of 3 and 5 mg/kg, with a percentage inhibition of 53% and 85%, (Figure 3B). The fraction with saturation of 60–90% showed a percentage of inhibition of 53–85% for neutrophils in comparison with the non-treated group and that injected with thioglycolate (Figure 3B).

The purification process proceeded with the fraction 0–30% saturation, which had exhibited the highest anti-inflammatory activity. This fraction was subjected to hydrophobic affinity chromatography using a C_4_ semi-preparative column, resulting in the separation of four distinct peaks: Peak 1: retention time (RT) 5.39 min, 5% TCA + 0.1% TFA; Peak 2: RT 21.75 min 38% TCA + 0.1% TFA; Peak 3: RT 29.9 min 46% TCA + 0.1% TFA; and Peak 4: RT 36.9 min and 52% TCA + 0.1% TFA, respectively. Only fraction three showed activity, with the percentage of inhibition ranging from 76 to 88%. A bioassay using this material at doses of 0.5, 1.0, and 3.0 mg/kg demonstrated a noteworthy inhibition from 72 to 80% of neutrophil migration into the peritoneal cavity of the mice. The total *AqGP* and reference drug dexamethasone, also evaluated in the assay, had inhibitory activities of 93% and 74%, respectively (Figure 3D).

**Figure 4:**
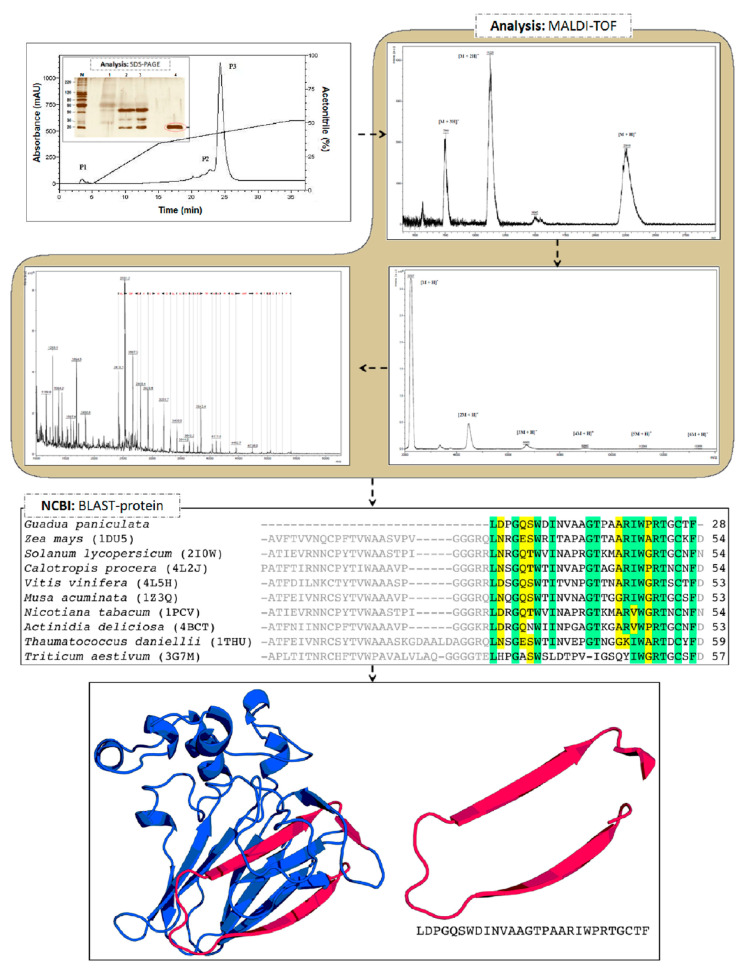
Steps for molecular identification of the bioactive compound isolated from *AqGP,* and classification as a thaumatin-like protein. Molecular mass profiles resulting from the first three fractionation steps. Where: M: molecular weight marker; 1: *AqGP*; 2: fraction 0–30%, precipitation with ammonium sulfate; 3: fraction 0–30%, precipitation with ammonium sulfate, dialyzed; 4: fraction with retention time of 29.9 min, obtained by HPLC C4 semipreparative column. The sequence alignment of the fragment retrieved from mass spectrometry with the PDB (Protein Data Bank) hits (the respective codes are between brackets) is: *Zea* *mays* [39], *Solanum lycopersicum* [40], *Calotropis procera* [41], *Vitis vinífera* [42], *Musa acuminate* [43], *Nicotina tabacum* [44], *Actinidia deliciosa* (Pavkov-Keller et al., unpublished)*, Thaumatococcus daniellii* [45], and *Triticum aestivum* [46]. Positions with conserved residues (80%) are highlighted in green; positions with similar residues (80%) are highlighted in yellow. The predicted three-dimensional structure of the chimeric zeamatin harboring the fragment of *AqGP*. The fragment is highlighted in pink. The structure shows virtually no modification, compared to zeamatin (RMSD = 0.2 Å). The model shows a discrete optimized protein energy (DOPE) score of 0.3; in the Ramachandran plot, 87.4% and 12% of residues are in favored and allowed regions, respectively; with a Z-score on ProSA of −5.91.

The *AqGP* and all its fractions previously selected by the neutrophil testing migration were submitted to denaturing electrophoresis. The proteins were separated in a sodium dodecyl sulfate polyacrylamide gel (12%) and were found to have molecular masses ranging from 20 to 50 kDa (Figure 4). A single band with a molecular mass around 20 kDa was observed for the single fraction purified by affinity chromatography using an analytical C_4_ column (Figure 4).

The protein purified using analytical C_4_ affinity chromatography and exhibiting anti-inflammatory properties was subjected to mass spectrometry analysis—MALDI-TOF, for a more detailed evaluation (Figure 4). The spectrum generated by analysis in reflector mode with mass-to-charge ratio (*m*/*z*) from 1000 to 3500 did not show any ionization product. However, the spectrum generated by analysis in a linear mode (with *m*/*z* ranging from 4000 to 24,000) exhibited three ions at different *m*/*z*, which seemed to correlate with the same macromolecule in different degrees of protonation. The largest ion was with *m*/*z* 22,400, and within this correlation the monocharged shape corresponded to [M + H]^+^; then formed a doubly-charged ion [M + 2H]^+^ at *m*/*z* of 11,200, and triply-charged form [M + 3H]^+^ at *m*/*z* of about 7400. The fraction was then subjected to MALDI in-source decay (ISD) using the matrix 1,5-diaminonaphthalene (DAN), and spectra were acquired with the target ion at *m*/*z* 22,400. The pattern of fragmentation obtained and the distances between each of the ions allowed us to obtain a de novo sequence of 28 amino acid residues (LDPGQSWDLNVAAGTPAARIWPRTGCTF). Isobaric residues (I/L and K/Q) were indicated after comparison with sequences from other organisms available in databases. This same sample was also subjected to analysis in the linear mode for larger *m*/*z* ranging from 20,000 to 140,000. The results again showed the presence of an ion at 22,400 and the presence of dimers, trimers, and other multimeric degrees. However, the molecule in its native form may not present this type of organization, which was possibly generated by the technique.

The 28 amino acid residues sequenced were then submitted to a local alignment against the NCBI protein database using BLASTp. The alignment results showed a high similarity of our sequence with a conserved region from thaumatin-like proteins, related to plant defense mechanisms against pathogens (Figure 4). The mature proteins from this family have ~200 amino acid residues, which is in agreement with the molecular mass observed in SDS-PAGE (Figure 4) and was confirmed by MALDI-TOF mass spectrometry. Comparing the sequence with the database showed a 57% similarity with the protein osmotin, which has been described in mouse studies as an anti-inflammatory agent for colitis [21], in addition to presenting a neuroprotective effect in excitotoxic diseases [47]. Osmotin has a high similarity in structure with adiponectin protein, which is well described in the literature due to its anti-inflammatory action [20].

The three-dimensional structure of thaumatins has three domains: domain I consists of a β-sheet; domain II, an extended α-helix associated with three shorter α-helices; and domain III, a β-hairpin turn with an extended loop (Figure 4). However, one thaumatin subfamily has been described that lacks domain II; the members of this subfamily are characterized as small thaumatin-like proteins [46]. In order to verify whether the identified thaumatin is a small thaumatin belonging to this subfamily, we performed fragment-based molecular modelling [48,49]. A zeamatin sequence was used as the basis for the fragment of *AqGP* and then this chimeric sequence was modeled. The fragment is near the N-terminal region of the protein in domain I and covers the beginning of domain III (Figure 4); this region has been predicted as a possible allergen epitope in other thaumatins, including Ban-TLP [43] and NP24-I [40]. The predicted three-dimensional structure, including the fragment, showed little modification in the RMSD (0.224 Å) in comparison with the zeamatin structure, indicating the absence of structural modifications. Although the fragment does not cover domain II, *AqGP* thaumatin does not necessarily belong to the small thaumatin subfamily. Given the similar anti-inflammatory properties of *AqGP* and osmotin [20,21], and the fact that the osmotin activity is related to domain II [20], it is possible that *AqGP* also contains domain II, which would characterize it as a thaumatin.

## 3. Materials and Methods

### 3.1. Plant Material

The rhizome of *G. paniculata* was collected in the city of Cristalina, Goiás state, Brazil. Data collection location coordinates were S 16°36,847′, 42,060″ W 47°36′49″ degrees. Initial botanical identification was performed by Dr. Dalva Graciano Ribeiro, from the Department of Botany, University of Brasilia, Brasilia (UnB), Brazil. A voucher specimen used in the identification was deposited in the UnB herbarium (protocol: 151468). The access to botanical material was registered in the Management System of Genetic Patrimony and Associated Folk Knowledge–SISGEN, Brazil (n°. A84EB12).

### 3.2. Plant Extract Preparation

The rhizome was washed with tap water, and immediately pulverized in a knife mill. The raw material was then dried at 50 °C in a forced ventilation kiln, and afterwards stored at room temperature in a cool dry place. The resulting powder was macerated in water 1/10 (*w*/*v*). The solution was maintained at 4 °C for 5 h and periodically stirred for 30 min intervals. The solution was then filtered through cheesecloth and centrifuged at 4 °C, 6000 g for 30 min. The pellet was discarded, and the supernatant was subjected to filtration using a 45 μm filter. The filtrate was lyophilized and stored at room temperature until further use [50,51].

### 3.3. Evaluation of Cell Viability

#### 3.3.1. Hemolytic Activity Test

Hemolytic activity was evaluated according to Aboudy and co-workers with some modifications [52]. Ten µL of *AqGP* with the concentrations of 0.32, 0.64, 1.28, 2.56, 5.12, and 10.24 mg/µL^−1^ was diluted in phosphate buffered saline (PBS), pH 7.4 was added to 190 µL of goat heparinized blood, washed and diluted to 8% (resulting in final concentrations of 0.32, 0.64, 1.28, 2.56, 5.12, and 10.24 mg/µL). After this procedure, the mixtures were incubated at 37 °C for 30 min under slow stirring. The material was centrifuged at 2000 g and incubated for 5 min at room temperature. The absorbance was monitored at 540 nm. The supernatant was then analyzed using a microplate spectrophotometer (Powerwave HT, Santa Ana, CA, USA). PBS and Triton™ X-100 (Sigma-Aldrich, St. Louis, MO, USA) 0.2% were used as negative and positive control, respectively. Three replicates were performed, and the procedure was repeated three times.

#### 3.3.2. MTT Assay

Murine NIH/3T3-fibroblast and MCF-7 human breast tumor cell lines were cultured in Dulbecco’s modified Eagle medium (DMEM; Gibco, Waltham, MA, USA) supplemented with 10% fetal bovine serum (Gibco), penicillin (100 U/mL) (Sigma-Aldrich), and streptomycin (100 mM/mL) (Sigma-Aldrich) and maintained at 37 °C in an atmosphere of 5% CO_2_. Cell cultures were maintained as described previously by Silva and co-workers [18]. Cells were removed (80% confluence) with the aid of a plastic carrier (TPP, Trasadingen, Schaffhausen, Switzerland). The cell concentration was adjusted to 1 × 10^5^ cells/mL. Cells were incubated in a 96-well (TPP) plate for 24 h, along with *AqGP* at various concentrations (0.32, 0.64, 1.28, 2.56, 5.12, and 10.24 mg/µL). The plate was incubated at 37 °C, 5% CO_2_. For the control treatment, the cells were incubated with PBS. After the incubation period, 150 μL (3-(4,5-dimethylthiazol-2-yl)-2,5-diphenyltetrazolium bromide) (MTT; Sigma-Aldrich) solution (5 mg/mL) diluted in 135 μL of DMEM (Gibco) was added to each well of the plate and again incubated for 3 h at 37 °C and 5% CO_2_ (in the dark). Subsequently, the microplate was centrifuged at 800 g for 5 min. The supernatant was removed, and formazan crystals were dissolved in 100 µL of DMSO (J.T. Baker, Center Valley, CA, USA) and stirred for 10 min at room temperature. The absorbance was monitored at 575 nm using a microplate reader (Bio-Tek, Winooski, VT, USA). Cell viability was expressed as a percentage compared to the untreated negative control cells and the positive control cells, which were treated with lysis solution (10 mM Tris-HCl, pH 7.4; 1 mM EDTA, 0.1% Triton X-100 (J.T. Baker, Center Valley, CA, USA)).

### 3.4. Animals

Male and female Swiss mice weighing 18–22 g (6 weeks) were obtained by the Bioterium of Universidade Catolica Dom Bosco (UCDB). Protocol number: 006/11. The animals were kept under conditions of controlled temperature (23–25 °C) and light/dark cycle, with free access to food and water.

### 3.5. Evaluation of Nociceptive Response

#### 3.5.1. Formalin Testsed

Nociceptive stimulation was performed by applying 20 μL of formalin (2.5%) (diluted in sterile saline) to the sub-plantar region of the right paw of mice (*n* = 5). The response to the stimulus was evaluated by observing the licking of the injured paw. Predetermined doses of *AqGP* were applied subcutaneously to the back of the animal 15 min before the nociceptive stimulus. The same procedure was performed with acetylsalicylic acid (Sigma-Aldrich), a proven acting analgesic. The number of licks was observed in two stages, first in the period 0–5 min after stimulation (step 1), and later in the period 15 to 30 min after stimulation (step 2) [33,53].

#### 3.5.2. Acetic Acid-Induced Abdominal Contortions

The mouse writhing test was performed based on the method of Koster et al. [54]. The mice were pre-treated with 0.9% saline, acetylsalicylic acid (100 mg/kg), *AqGP* 1.0, 5.0, and 10 mg/kg (s.c). Acetic acid (0.8%, 100 μL, i.p.) was given 15 min after treatment. Immediately after the injection of a nociceptive stimulus, the number of abdominal contortions, characterized by abdominal rotation and total stretching of the hind paws were counted for 30 min.

### 3.6. Anti-Inflammatory Assay: Neutrophil Migration

The inflammatory process was performed as describe by Roriz and colleagues [55] and induced by application of 500 µL thioglycolate (Merck, Kenilworth, NJ, USA) 4% per animal (*n* = 5), in the intraperitoneal region. After 6 h of phlogiston stimulation, the animals were euthanized in a CO_2_ chamber. Subsequently, an incision was made to expose the animal’s intraperitoneal region, which was washed with 3 mL of isotonic EDTA solution (32% (*w*/*v*)). An aliquot of 20 μL of this washed material (exudate) was collected and mixed with 380 µL of Turk blue to achieve the overall cell count using a Neubauer chamber. The cell exudates were precipitated by centrifugation at 2800× *g* for 2 min and used for smear preparation. After this process, the material was stained with Quick Kit Panoptic (Laborclin, Rio Preto, SP, Brazil) and used for differential counts. For the control, 500 µL of 0.9% saline solution (*w*/*v*) was administered intraperitoneally. After this procedure, the material was submitted to the procedures described above. To evaluate the effect of *AqGP* and its fractions on the migration process, predetermined doses of 100 µL were applied subcutaneously to the back of the animal, 15 min before the inflammatory stimulus by thioglycolate (Merck). For quantitative analysis of the material, the same procedure was performed with anti-inflammatory Dexamethasone (Sigma-Aldrich) (0.5 mg/kg).

### 3.7. Anti-Inflammatory Bioassay Guided Fractionation

#### 3.7.1. Dialysis Fractionation

The lyophilized plant extract was dissolved in 10 mL of distilled H_2_O and then fractionated by dialysis against distilled H_2_O using a membrane (Spectrum Laboratories Inc, Rancho Dominguez, CA, USA) of pore size of 3.5 kDa. Dialysis processes were maintained at 4 °C for 48 h under slow agitation and periodic changes of distilled H_2_O, always in a proportion of 1/200 H_2_O. The two fractions obtained by this process (internal dialysate molecules >3.5 kDa and external dialysate molecules <3.5 kDa) were lyophilized and then subjected to biological evaluation of anti-inflammatory action by neutrophil migration [56].

#### 3.7.2. Fractionation by Ammonium Sulfate Precipitation

The internal dialyzed fraction, containing molecules larger than 3.5 kDa, was subjected to precipitation by ammonium sulfate (Sigma-Aldrich). This fraction was quantified by Bradford (Bio-Rad Laboratories, Hercules, CA, USA) [57] and diluted in water in a ratio of 1 mg/mL. After this procedure, the material was precipitated by saturation in 0–30%, 30–60%, and 60–90% ammonium sulfate, according to the table available in [58]. At each stage of saturation, the material was maintained at 4 °C for 4 h, and subsequently centrifuged at 7500 g for 15 min. The precipitate was diluted in distilled H_2_O and dialyzed against a membrane of 3.5 kDa pore size for desalination. The precipitated fractions and the non-precipitated fraction from 60–90% were subjected to biological evaluation of anti-inflammatory action by neutrophil migration.

#### 3.7.3. Fractionation by High Performance Liquid Chromatography

The fraction saturated with 0–30% ammonium sulfate was desalinized, lyophilized, and resuspended in 0.1% TFA (J.T. Baker, USA), and further purified using HPLC (Shimadzu, Japan) on a Vydac™ C4 semi-preparative column (Grace, Bannockburn, IL, USA), with a nonlinear acetonitrile (J.T. Baker, USA) gradient (5–95%) at a flow rate of 2.5 mL/min. Sample elution was monitored at 216 and 280 nm. The HPLC fraction obtained in the retention time of 29.9 min (500 µg) was applied onto a HPLC and eluted with a linear gradient (5–95% acetonitrile), at a flow rate of 1 mL/min using an analytical C_4_ column (Grace^®^, Columbia, USA). All fractions were lyophilized and submitted to biological assay to evaluate the anti-inflammatory effects by neutrophil migration [56].

### 3.8. Chemical Identification Methods

#### 3.8.1. SDS-PAGE Analysis

The selected fractions from the purification steps (*AqGP*, internal dialysate, 0–30% ammonium sulfate saturation, and the peak resulting from the chromatography step), containing 40 µg each were resuspended in 15 μL of 1× sample buffer and then boiled for 5 min. Samples were then loaded in SDS-PAGE 12% [59]. The proteins were separated by applying a voltage of 15 mA for the concentration gel and 25 mA for the separating gel. After the electrophoresis process the gel was stained with Coomassie brilliant blue (Thermo Scientific, Waltham, MA, USA) and silver (Sigma-Aldrich).

#### 3.8.2. Mass Spectrometry Analysis by MALDI-TOF

The fractions were diluted in 10 µL of Milli-Q H_2_O and an aliquot (1 µL) was mixed with saturated solution of acid matrix α-cyano-4-hydroxycinnamic acid (Bruker Daltonics, Bremen, Germany) in a 1:3 ratio (sample:matrix *v*/*v*). The mixture was applied onto a steel plate for MALDI-TOF and inserted into a UltraFlex III MALDI-TOF mass spectrometer (Bruker Daltonics) after crystallization at room temperature. Data were collected by FlexControl software version 3.0, and the mass spectra were acquired in linear and reflector modes, using external calibration. For the linear mode, the samples were analyzed utilizing a range between *m*/*z* 4000–25,000 and *m*/*z* 20,000–140,000. The calibrants used were the Protein Calibration Standards I and II (Bruker Daltonics). For the reflector mode, the samples were analyzed in a range of *m*/*z* 1000–4000. The calibrant used was Peptide Calibration Standard I (Bruker Daltonics). Intact protein fragmentation was accomplished directly in the in-source decay mode (ISD) with a 1,5-diaminonaphthalene (DAN) matrix (Sigma-Aldrich). This mode, comprising a range of *m*/*z* 1000–6000, allowed for manual interpretation of the spectrum from the ions c series, which enabled the elucidation of a partial primary structure for the intact protein. The sequences obtained were submitted to a local sequence alignment search against the NCBI protein database using BLASTp [60].

### 3.9. Fragment-Based Modeling

The fragment retrieved from mass spectrometry analysis was submitted to a PSI-BLAST [60] search against the protein data bank, through four iterations, when no new sequences were retrieved. The sequences were aligned with the fragment by means of ClustalW2 [61]. The molecular model was constructed according to fragment-based modelling [48,49], with minor modifications. A chimeric sequence was constructed, introducing the *G. paniculata* fragment into the zeamatin sequence [39]. Modeller 9.17 [62] was used to construct 100 models, using the zeamatin structure (PDB ID: 1DU5) as the template. The models were constructed using the default methods of automodel and environ classes from MODELLER. The final models were selected according to the discrete optimized protein energy score (DOPE score). This score assesses the energy of the model and indicates the best probable structures. The best models were evaluated through PROSA II [63] and PROCHECK [64]. PROCHECK checks the stereochemical quality of a protein structure through the Ramachandran plot. Good quality models are expected to have more than 90% of amino acid residues in the most favored and additional allowed regions, while PROSA II indicates the fold quality. The root mean square deviation (RMSD) between the zeamatin structure and the chimeric structure was calculated by using a 3dSS server [65] by means of STAMP algorithm [66]. Structure visualization was done in PyMOL [67]

### 3.10. Statistical Analysis

Data are presented as the mean ± SD. Comparisons between two groups were made by an unpaired t test. Comparisons between more than two groups were submitted to one-way analysis of variance (ANOVA) followed by Bonferroni correction. Values of *p* < 0.05 were considered statistically significant. GraphPad Prism software v 6.0 (GraphPad Software, La Jolla, CA, USA) was used for all statistical analyses.

## 4. Conclusions

In conclusion, we demonstrated the anti-inflammatory and analgesic properties of *G. paniculata* aqueous extracts. A thaumatin-like protein isolated from these aqueous extracts appears to act as the active principle of such activities. This fact is reinforced by the similarity to osmotin, another thaumatin-like protein, which presents analgesic effects. These data suggest that the isolated protein either alone or together with other molecules is responsible for the anti-inflammatory activity of the *G. paniculata rhizome* used in Brazilian folk medicine. Despite the fact that thaumatins may present an allergenic epitope, our data indicated that the thaumatin-like protein from *G. paniculata* is safe to be used as a drug. However, further studies are needed for a better characterization of this compound, regarding its primary structure and characterization of its epitopes and the determination of the small active fragments. Nevertheless, the identification of anti-inflammatory and analgesic properties from a thamatin-like protein could expand the repertory of biological activities from this protein family. Moreover, the identification of such a potential drug from *G. paniculata*, reinforces the folk medicine as a resource for bioactive molecules.

## Data Availability

The data presented in this study are available on request from the corresponding author.

## Sample Availability

Samples of the *G. paniculata* rhizome are available from the authors.
